# 

**Published:** 1894-04-07

**Authors:** 


### The Hospital

AN INSTITUTIONAL JOURNAL OF

THE MEDICAL SCIENCES AND HOSPITAL ADMINISTRATION,

INCLUDING

A SPECIAL SUPPLEMENT FOR NURSES.

EDITED BY

HENRY C. BURDETT.

SURGEON GEN'S OFFICE
150280
LIBRARY.

IN TWO VOLUMES ANNUALLY.

**VOLUME XVI.**

April to September, 1894.

London:
PUBLISHED BY THE SCIENTIFIC PRESS (LIMITED), AT THEIR OFFICE, 428, STRAND, W.C.

MDCCCXCIV.

W1
N0L9X

### INDEX TO VOL. XVI., 1894.

Addenbrooke's Hospital, 73

Advancement of Science by Dining, 95

Ainger's (Canon) True Physician, 385

Alcohol as a physiological peacemaker, 95

Alcohols, Caustic or the Ethylates, 351, 387

Alexandra Hospital (A.H.), 310

Anderson v. Cook, 117

Annotations (*Under the various sub-titles*).

Around the Hospitals (*See also under the names of the various hospitals*), 2, 24, 46, 68, 90, 112, 134, 178, 200, 222, 266, 288, 310, 330, 350, 368, 386, 422, 440, 458, 476.

Artificial respiration in Chloroform Collapse, 71, 113

Assurance, Medical, Defects of, 105

Aspects of Medical Science, Some, 206

Asylum of the World, The, 497

Asylums in India (W.), 40, 346

Asylums, Unlicensed, 461

Autopsy, A Bureau of, 478

Babies of Battersea, The, 389
Bacillus Diphtheriæ in Cow's Milk, 315
Bacteriology in the Diagnosis of Diphtheria, 405
Barrack Children. An Object Lesson, 353
Berkshire Hospital, Royal (A.H.), 46
Bideford Infirmary (A.H.), 386
Bicycle, The Dangerous, 425
Birmingham Hospital Saturday Fund (W.), 127, 149, 171, 194, 213, 237, 258
Birmingham, Royal Visit to (W.), 487
Book World of Medicine and Science, The (books, magazines, and pamphlets reviewed or noticed, and articles relating to)—
Baker. New Guide to Bristol and Clifton, 365
Books for the Sick, 65
Bristowe. Legal Hand-book, 65
Burdett. Hospital and Charities Annual, 1894, 63
Central Blatt für Chirurgie, 241
Crede and Leopold. Examination of Lying-in Women, 87
Crook. Cimiez: Its Health and Climate, 175
Curtice. Index to the Times, etc., 383
Donald. Introduction to Midwifery, 21
Ellis. Health Resorts of Northumberland and Durham, 327
Fellow of the Chemical Society, A. Beginner's Guide to Photography, 437
Goodhart. Common Neuroses, 43
Jadroo. Disease and Race, 523
Martin, Electricity and Diseases of Women, 175
Martindale. Analysis of 12,000 Prescriptions, 437
Medical Annual, 1894. 175
Mountain, Moor and Loch, 383
Official Year-Book of Scientific and Learned Societies, 383
Oliver. Student's Handbook of Botany, 109
Parkes. Infectious Diseases, 109
Pepper. Theory and Practice of Medicine, 153
Periodicals, 43
Pharmacopeia of the National Hospital for Diseases of the Heart, 383
Photography. Beginner's Guide to, 437
Prescription Books, 365
Robottom, Travels in Search of New Trade Products, 365
Russell. Epidemics, Plagues, and Fevers, 65
South African Empire, The, 383
Sturmer. Cyclist's Year-Book, 1894, 175
Therapeutics of Literature, 65
Trevor-Battye, Nature, Wild Sport, and Humble Life, 327
Vines. Text-Book of Botany, 21
Weir-Mitchell. Characteristics, 87
Williams. Æro-therapeutics, 43
Willing. British and Irish Press Guide, 327
Wilson, W. *See Crede and Leopold*
Zeitschrift für Hygiene, 241
Brain, Localisation of Sensation in the, 137
British Medical Association at Bristol, 391, 413, 431, 449, 467, 485
British Lying-in Hospital, The (A.H.), 508
British Medical Benevolent Fund, The, 183
Bromal Hydrate, 311
Bromine in Therapeutics, Role of; and Bromal Hydrate, 311
Buda-Pesth, International Congress at, 493.
Buda-Pesth, Reminiscences of, and the International Congress, 520

Canadian Hospitals (W.), 470
Chelsea Hospital and Dr. Parkes, 29
Chelsea Hospital for Women (W.), 349, 361, 364
Chester General Infirmary (A.H.), 440
Cholera, Koch on, 507
Chloroform Collapse. *See Artificial Respiration.*
Civilisation, Place of Medicine in, 67
City of London Hospital for Diseases of the Chest (A.H.), 476
Clark, Sir A., Memorial to, 115, 129
Clinical Research Association, 479
Cocoas and Chocolates (W.), 433, 452
Commercial Medicine. A prophecy fulfilled, 293
Confessional. The Medical, 407
Construction Notes (W.), 19, 42, 64, 84, 152, 197, 262, 306, 326, 350, 382, 399, 435, 454, 490, 521
Consumption and Infection, 5
Continental Notes from a Holiday Correspondent, 263 307, 347, 401, 404, 473
Copenhagen, Queen Louise Hospital (A.H.), 289
Cornwall, A "Bitter Cry" from, 271
Correspondence. [*Editor's Letter Box*]
Corstorphine, Royal Infirmary (W.), 379
Costliness of Modern Treatment, The, 439
Creasote. Effects of Creasote upon Tuberculosis, 511
Cumberland Infirmary (A.H.), 24
Cure, A New, 497
Cure, An Unexpected, 139
Cure. Is Cure Superfluous? 315
Curiosities of American Lunacy, 371

Darlington Hospital (A.H.), 46
Dental Hospital, Leicester Square (A.H.), 266
Derby Sets an Example, 315
Derbyshire Royal Infirmary, The (W.), 325, 343
Dermatological Society, A New, 51
Dermatology and General Medicine, 205
Devonshire Hospital, Buxton (A.H.), 178
Diabetes, Dr. Pavy's Researches on, 352
Diet and Stupidity, 95
Diphtheria at Leavesden, 51
Diphtheria (School) in Large Towns, 479
Diphtheria, Bacteriology in the Diagnosis of, 405
Diphtheria Problem, The, 511
Diphtheriæ Bacillus in Cow's Milk, 315
Do Doctors Feel? 7
Doctor Afloat, The, 329
Doctors in Council. 367
Doctors. Should Doctors Trade? 227
Drugs, *See New Drugs, &c.*

Eastbourne, Princess Alice Hospital (A.H.), 458
Edinburgh, Convalescent Home of the Royal Infirmary (W.), 379
Editor's Letter-box, 87. 196, 213, 236, 258, 280, 322, 346, 406
Educational Treatment of Idiots, &c., 92, 332, 406
Epsom. What Epsom does for doctors, 335
Ethics and Administration, 435
Ethylate, Potassium, 441
Ethylates, The, 351, 387
Eucalyptus Oil, 236

Famous Poisoners in Fiction, 26, 333
Field of Science, 6, 72, 138, 182, 226, 270, 334
Finance. *See Hospital Finance*
First Century of English Medical Literature, The, 28, 50, 136, 180, 224, 267, 313, 442
Foreign Hospital Intelligence (W.), 17
Foster (Professor) and Scientific Pin-pointers, 7
Foster (Professor) on the Organisation of Science, 38

General Medical Council, The (W.), 155, 193
Germs not Pathological, 243
Gladstone (Mr.) on Medicine, 111
Gladstone's (Mr.) Sight, 353
Golf, 461
Golf Cure for Insomnia, 479
Guardians and Nurse Gillespie, 271

Harrogate, Royal Bath Hospital (A. H.), 368
Hastings and East Sussex Hospital, (A. H.), 2
Health and Holiness, 424
Heat Stroke in England, 335
Herbal Simples, History and Capabilities of, 244, 292
Holiday Season, The, 425
Holiday Thoughts for Health Makers, 457
Homœopathic Hospital, London (W.), 282
Home Hospitals Association (A.H.), 200
Homes of England, The, 48
Hospital Administration (W.), 149, 171, 237, 435, 469
Hospital Construction (W.) 282, 325, 343, 379
Hospital Festivals, etc. (W.), 19, 41, 61, 85, 109, 129, 151, 173, 194, 239, 262, 284, 306, 325, 363, 382
Hospital Finance (W.), 39, 83, 105, 259, 303, 519
Hospital Laundry, The (W.), 489, 521
HOSPITAL SUNDAY SPECIAL SUPPLEMENT, 215
Hospital Sunday Conversazione at the Mansion House (W.), 239
Hospital Sunday Fund, Metropolitan (W.), Particulars of Awards for 1893, 381
Hospital Sunday and Saturday Funds, Early History of the (W.), 451
Hospitals, Canadian (W.), 470
Huddersfield Infirmary (A.H.), 458
Humanity of Medicine, The, 199
Huxley on Head and Heart, 51
Hydrides and Hydrates, Researches on, 157
Hydrides, Further Notes on the, 245
Hygiene in University Education, 415

Idiots, &c., Educational Treatment of, 92, 332, 406
India, Asylums in (W.), 40, 346
India, Hospitals in (W.), 305, 435
Indigestion, That Vile, 293
Influenza, A Theory of Cause and Cure, 389
Insane, *See Story of the Insane*
Insane. The After Care of the, 443
Insanity, Can Doctors prevent Insanity? 249
International Congress at Buda Pesth, The, 493
International Medical Congress at Rome, 15, 38
Irish Lunacy Blue Book (W.), 17
Isle of Man General Hospital, Noble's (A. H.), 508

Jewish Incurables, Home and Hospital for (A. H.), 476
Journalists. Should Journalists Marry, 29
Judges and Justice: Anderson v. Cook, 117
Justice for Doctors, Some, 183

Koch on Cholera, 507

Labour and Health, 478, 510
Leeds General Infirmary (A. H.), 24
Lighthouse Keeper's Grievance, The, 511
Lister Portrait, The 139
Liverpool Royal Infirmary (A. H.), 368
Lives we have bid to be, The, 160
Localisation of Sensation in the Brain, 137
London, A Teaching University for, 1
London Fever Hospital (A. H.), 350
Louth Charitable Infirmary (A. H.), 68
Lunacy, Curiosities of American, 371
Lunatic's Relatives, A, 363
Lungs of London, The, 407

M.O.H.? Who Supervises the, 335
Malaria, Modern Views of, 27, 94 (*and see p.* 196)
Man, The Study of, 225, 269
Medical Aid Associations, 177
Medical Ambitions, 389
Medical Education in the Provinces, 518
Medical Education in Scotland, 517
Medical Confessional, The, 407
Medical Literature, English. *See First Century*
Medical Progress, The Summits of, 309
Medical Progress and Hospital Clinics, *and also* Progress in Surgery
Abdominal Surgery, 58
Abscess, Sub-diaphragmatic, 124
Angular Deformity of the Spine, Treatment of, 317
Appendicitis, 256
Bone, Joint, and Orthopædic Surgery, 358, 377, 430, 464
Breast, Cancer of the, 340
Cancer, Operative Treatment of, 340
Carbolic Acid, Simple Uses of, 252
Children, Diseases of, 338, 357, 375
Chronic Catarrh of the Middle Ear, 355, 373, 463, 481
Clubfoot, 55

Medical Progress and Hospital Clinics—*continued.*
Congenital Displacement of the Head of the Femur and its Treatment, 119
Constipation, Habitual, Treatment of, 446
Cranial Surgery, 146
Cysts and Fistula in Connection with a Persistent Thyro-glossal Duct, 120
Dermatology, Periscope of, 396, 410
Dermatology, Progress in, 143
Dilatation of the Stomach, 33
Ear, Chronic Catarrh of the Middle, 355, 373, 463, 481
Ear, Diseases of the, 274, 295, 319, 337, 513
Ear, Gravity of any Discharge from the, 251
Electricity and its Use in Medicine, 9, 31
Exophthalmic Goitre and its Relations to Diseases of the Thyroid Gland, 165, 186
Eye, Diseases of the, 81
Femur. *See Congenital Displacement*
Fevers and Infectious Diseases, 232
Fibro-Myotamous Tumours of the Uterus Producing Bladder Symptoms, 445
Fractures, Treatment of (Simpler Methods), 427
Gastric Ulcer and its Treatment, 296
General Surgery, 301, 341, 359
Genito-Urinary Surgery, 412
Goitre, Exophthalmic, 165
Gonorrhœa and its Effects, 210
Gout in Hospitals, 98
Hæmorrhage during Pregnancy, Causes of, 97
Hæmorrhoids, 15
Heart Disease (Chronic) Complicating Pregnancy and Labour, 208, 229
Heart and Vascular System, Diseases of, 428, 483, 515
Hernia, 255
Intestines, Surgery of the, 188, 234
Intra-Uterine Syringing, 142
Intussusception, Acute, 207, 231
Iritis (Acute), Treatment of, at London Hospital, 120
Joint Disease, Symptoms in, 76
Joint Surgery, 358, 377, 430
Kidney Diseases, 298
Liver and Gall Bladder (Surgery), 276
Loretine, 37
Lungs and Pleura, Surgery of, 123, 466
Malignant Growths, 36, 278
Myoma Uteri and its Treatment, 190
Nervous Diseases, Progress in, 211
Nervous System, Diseases of the, 166
Œsophagus and Stomach, Surgery of the, 187
Orthopædic Surgery, 358, 377, 430, 464
Pancreas and Spleen, Surgery of, 277
Paracentesis of the Various Cavities, Notes on Performing, 514
Peritonitis, 79
Peritonitis (Pelvic), Treatment of, 163
Pleura. *See Lungs*
Pleurisy, 120
Pneumonia, 12
Puerperal Septicæmia, Intra-Uterine Syringing in, 142
Pulmonary Cavities, Treatment of, 102
Pulmonary Tuberculosis, 35, 56, 77
Rectal Surgery, 14
Rectum, Innocent Tumours of the, 395
Rectum, Malignant Tumours of the, 409
Renal Affections, 99
Renal Medicine, 446
Renal Surgery, 300
Scarlet Fever, 11, 320
Scorbutic Disease in Children, 273
Small-pox, 11
Spinal Caries, 317
Stomach. *See Dilatation and Œsophagus*
Surgery, General, 301, 341, 359
Genito Urinary, 412
Orthopædic, 358, 377
Renal, 300
Temperature Charts, 75
Tinea Tonsurans, 55
Tuberculosis, 141
Tuberculosis, Surgical Treatment of, 102
Typhoid Fever, 253, 275
Uric Acid, How to Avoid Troubles Due to, 53
Urine in Typhoid Fever, 185
Vascular System, Diseases of the, 428, 483, 515
Wounds, Treatment of, 297

Medical Protection, 249
Medical Science, Practical Aspects of, 70, 158
Medical Science, Some Aspects of, 204, 268, 292, 312, 388
Medical Students' Vade Mecum, The, 499
Medicine in Civilisation, Place of, 67
Medico-Psychology and Psychiatry, Modern, 25, 69, 114, 159, 203, 247, 423, 496
Metropolitan and National Nursing Association, 107
Metropolitan Hospital Sunday Fund, 381
Middlesex Hospital Festival Dinner (W.), 61
Miller Hospital, Greenwich (A.H.), 494
Modern Progress in Ophthalmic Medicine and Surgery, 3, 47, 91, 135, 179, 223, 289, 331, 369, 459

National Hospital for the Paralysed and Epileptic (A.H.), 330
New Appliances and Things Medical, 37, 60, 82, 104, 125, 147, 170, 234, 257, 279, 302, 321, 360, 378, 432, 450, 468, 486.
New Drugs and Preparations, 59, 82, 103, 125, 169, 192, 257, 321
Noble's Isle of Man General Hospital (A.H.), 508
North London Hospital for Consumption (A.H.), 494
North-West London Hospital (A.H.), 386
Notes and News, 8, 30, 52, 74, 96, 118, 140, 162, 184, 206, 228, 250, 272, 294, 316, 336, 354, 372, 390, 408, 426, 444, 462, 480, 498

Oldham Infirmary (A.H.), 200
Opium or Alcohol, 117
Ophthalmic Medicine and Surgery. *See Modern Progress.*
Outrage at the British Medical Association, An, 371

Paranoiacs at Chicago, 139
Patients' Payments (W.), 39
Pauper's Pipe, The, 461
Physician, Canon Ainger's True, 385
Physician to Physicians, A, 265
Physiology and Collectivism, 353
Physiology and Property, 7
Place of Medicine in Civilisation, The, 67
Plagues of the Bible, The, 246
Playgrounds for Little Players, 371
Poisoners. *See Famous Poisoners.*
Poisoning, A Check on Public, 293
Political Insanity, 183
Poplar Hospital for Accidents (A.H.), 310
Potassium Ethylate, 441
Practical Aspects of Medical Science, 70, 158
Practical Books for Hospital Workers, 63
Practical Departments (W.), 18, 40, 64, 84, 194, 305, 324, 363, 380, 399, 433, 452, 471, 489, 521
Practical Points (W.), 42, 282, 325, 522
Practice, Old Fashioned, 227
Preventive Family Medicine, 117
Princess Alice Memorial Hospital, Eastbourne (A.H.), 458
Provident Dispensary Failure, A, 161
Psychiatry. *See Modern Medico-Psychology*
Psychologist in History, The, 49, 370, 477
Public Schools: Facts for Parents and the Press, 73

Queen Louise Children's Hospital, Copenhagen (A.H.), 289
Queen's Hospital, Birmingham (A.H.), 200

Radcliffe Infirmary, Oxford, 407
Rational Therapeutics and Therapeuticians, 133
Reactionary Medicine, 221
"Religio Medici," 161
Richardson's (Sir B. W.) Researches, 201
Richmond Royal Hospital (A.H.), 112
Roof Gardens for Londoners, 443
"Royal and Ancient Game" at St. Andrews, The, 461
Royal Bath Hospital, Harrogate (A.H.), 368
Royal Berkshire Hospital (A.H.), 46
Royal Free Hospital (A.H.), 386
Royal Hospital for Diseases of the Chest (A.H.), 134
Royal Hospital, Richmond (A.H.), 112
Royal Sea Bathing Infirmary (A.H.), 222
Royal Surrey County Hospital (A.H.), 24

St. George's Hospital (A.H.), 476
St. Mary's Hospital (A.H.), 178
St. Thomas's Hospital Medical School, 235
St. Thomas's Hospital Medical School (W.), 260
Salford Royal Hospital, 288
Salisbury's (Lord) Address, 403
Sanitation, Dry, 271
School Fevers, 45
Science. *See Medical Science*
Scientific Method, The, 29
Scraps and Gleanings, 20, 42, 64, 87, 108, 152, 174, 197, 240, 262, 303, 326, 346, 364, 382, 400, 436, 454, 472, 490, 522
Self-poisoning, Unconscious, 249
Should Doctors Trade? 227
Should Journalists Marry? 20
Sick Children, Hospital for (A.H.), 266
Sieveking (Sir E.) on Out-patients, 205
Small-pox and Vaccination at Leicester, 205, 491
Soap. The Place of Soap in Strikes, 443
Social Pathology and Education, 5
Sociology, Modern, 473, 510
Sociology, Studies in Applied, 4, 48, 93, 160, 202, 248, 290, 314
Southport Convalescent Hospital (A.H.), 68
Southport Infirmary (A.H.), 330
Stammering, The Cure of, 93
Story of the Insane from Year to Year (W.), 19, 150, 281, 304, 323, 345, 434, 453, 472, 489
Street Collections, 455
Student Life in the Sixteenth Century, 495
Student of Medicine, The, 241, 263, 285, 307, 327, 347, 365, 383, 401, 419, 437, 455, 473, 491, 522
Suffolk General Hospital (A.H), 90
Suicide. Why is Suicide on the Increase? 161
Suicide, The Prevention of, 227
Summits of Medical Progress, The, 309
Sunderland Infirmary (A.H.), 422
Supplement, Special Hospital Sunday, 115
Surgeons (Operative), The Great Untaught, 421
Surgery, Progress in. *See under Medical Progress*
Surrey County Hospital, Royal (A.H.), 24
Sussex County Hospital (A.H.), 179

Teaching University for London, A, 1
Therapeutics. *See Rational Therapeutics*
Toxic Neutralisations, On, 509
Trade. Should Doctors Trade? 227
Typhus Fever and Difficulties in Diagnosis, 181

Ubi Est Morbus? 23
Undignified French Dignities, 497
Unexpected Cure, An, 139
University College Hospital (A.H.), 222
Untaught, The Great, 421

Vacancies and Appointments, Medical, 22, 44, 66, 88, 110, 132, 154, 176, 198, 241, 263, 285, 307, 327, 347, 365, 383, 401, 419, 437, 455, 473, 491, 522
Vaccination, A New Research? 287
Vaccination and Compulsory Vaccination, 475
Vaccination. *See Small-pox*
Village Doctor: New Style, A, 73

Westminster Hospital (A.H.), 266
Work for the Duke of York, 425
Workers. How Workers Live, 202, 248, 290, 314
Workshop, The Institutional, *See*
Asylums in India
Birmingham Hospital Saturday Fund
Chelsea Hospital for Women
Construction Notes
Foreign Hospital Intelligence
General Medical Council
Hospital Administration
Hospital Festivals
Hospital Finance
Hospital Sunday Fund
Irish Lunacy Blue Book
Practical Books
Practical Departments
Practical Points
Story of the Insane from Year to Year
Wrexham Infirmary (A.H.), 134

York, Work for the Duke of, 425

150280

### THE SPECIAL SUPPLEMENT FOR NURSES.

Accident, A sad, clxv
Acqui and its Mud Baths, lxv
Advantages and Privileges of a Trained District Nurse and the Duties of the District towards her, liv
Ambulance Work in Paris, clxiii
America, Notes from, xxix
American Letter, Our, lxvii, clv, ccv
American Sisters, Our, clix
Amongst the Children: Isolated or Caged? clxxxiv
Annual Leave, Our, lvii
Appointments, x, xxvi, xxxviii, xlvi, lv, lxvii, lxxix, lxxxiv, c, cv, cxiv, clix, clxx, clxxx, clxxxvi, cxcvi, ccxv, ccxliv
Appointments, Minor, x, xviii, xxviii, xxxiii, xlix, lvi, lxx, lxxxvii, c, cx, cxxv, cxxxiii, clix, clxxx, clxxxv, ccix, ccxxv, ccxxxvii, ccxliv
Architects, Women, ccviii
Assistance, A Case for, cxxvii, cxxxiv, clviii, clxvii, clxxvii, clxxxviii, cxciv, cciv, ccxviii, ccxxv, ccxxxii, ccxxxvi, ccxliv
Asylum Attendants, Certificated, lxxxvi
Asylum Notes (Ireland), clv
Australia, Ladies in, lxiv

Bedford, Opening of the Nurses' Home, iv
Bedford Workhouse, cxcvi
Bequest to Nurses, A Generous, xx
Bishop Auckland, The Workhouse Infirmary at, clxvii
Book World for Women and Nurses. *On Extra Pages*
Brazil, English Nurses in, lxxxvi, xcvi
Brazil, Hospitals in, ccxxiii
British Home for Incurables, Royal Visit to the, cxxxv
Busy Quarter, A, clxiii

Café Chantant, A successful, c
Caithness, ccix
Canada, A Visit to, lxviii, lxxv
Carbolic Acid, Death from, v
Character Sketches from a Lunatic Asylum, cxcv, ccvii, ccxxxi
Chester Workhouse Infirmary, clxxiv
Children. *See Amongst the Children*
Children's Country Holidays' Fund, cxvii
Cholera Precautions, cxcvi
Clapham School of Midwifery, vi
Cookery and Food Association, The Universal, clxiv
Craig Colony for Epileptics, cxxvi

Dartford, Enthusiasm at, clxx

Death in Our Ranks, l, ccxxxi

Diet in Disease, ccxxix, ccxxxv, ccxli, ccxlvii

Disinfecting Rooms, Hints on, cxcvii

Disinfection, Hints on, clxxvii

District Nurse. *See Advantages*

District Nurses, lxxxvii

Droylesden, A Trained Nurse for, xxxv

Duty versus Danger, lxxxiii

Emergency, cxviii
Enteric Fever in Children. *See Nursing*
Entertainments, cxx, clxvii, clxxvii
Epileptics, Craig Colony for, cxxvi
Erratum, ccix
Everybody's Opinion, x, xx, xxx, xxxix, xlviii, lxx, lxxx, xc, c, cx, cxx, cxxx, cxxxix, clx, clxx, clxxix, clxxxvi, cc, ccx, ccxx, ccxxvi, ccxxxii, ccxxxviii, ccxlix
Examinations, Our, xix, lxxvi, cviii, clv
Exeter City Asylum, cxxvi
Exhibitions and Entertainments, xcviii

Garden Fête, A, ccxvi
Germany, Notes from, cxxv, clv, clxxxiv, ccv, ccxlviii
Gillespie, Sentence Passed on "Nurse," cxxviii
Great Northern Central Hospital, xxxviii
Guy's Hospital, The New Laundry Hostel, ccxlix

Hampton (Miss) in England, clxxv
Holidays, Children's Country Fund, cxvii
Holland, Notes from, cxxxviii
Home for the Dying, A, cxxxvi
Hong Kong, The Plague in, clxxvi
Hospital for Sick Children, xxxviii
Hospital, A Spanish, xxviii
Hospital Treatment, xxxvi, xlvi
"Hospital" Convalescent Fund, The, cxl

Imagination and Emotion, clvi.
India, Notes from, ccvi
India, Nurses for Civilians in, cviii
Indian Nursing Service, xxiv
Insane, Character in the, cxv
Irish Hospitals, ccxlii

Japan, Holidays in, ccvii, ccxviii, ccxxiv
Jewish Incurables, Home and Hospital for, clxxviii
Jubilee Institute for Nurses, Queen Victoria's, lvi, clviii

Kew, Nursing Home at, xiv

Labrador, English Nurses on the, ccviii
Labrador, Nursing on the, ccxxxvii
Lectures at St. George's Hospital, v
Lecturing, On, lxxvi
Lepers at Robben Island, ccvii
Liskeard, clxxxvi
London Obstetrical Society, clxxx

Marriage, cliv
Marsden, Miss Kate, ccxvi
Maternity Work in U.S.A., lx
Matrons' Council, The, clxv
Mechanism of Movement, lxxxiv, xciv, civ, cxiv, cxxvii, cxxxiv, cliv, clxiv (*and see p.* cxxxix)
Medico-Psychological Association, lxx
Melbourne, News from, xcv
Melbourne, Notes from, xxvii, cxviii
Midwives and Trained Nurses' Club, The, clxxxiv
Mile End, The Pride of, ccxxx
Minor Appointments. *See Appointments, Minor*
Morningside Asylum, cx
Muses' Looking Glass, The, ix, xxix, xl, xlix, lix, lxix, lxxix, lxxxix, xcix, cix, cxix, cxxix, clxix, clxxxix, cxcix, ccxix

National Home Reading Association, clvii
New York Teachers' College, lx
News from the Nursing World, i, xi, xxi, xxxi, xli, li, lxi, lxxi, lxxxi, xci, ci, cxi, cxxi, cxxxi, cli, clxi, clxxi, clxxxi, cxci, cci, ccxi, ccxxi, ccxxvii, ccxxxiii, ccxxxix, ccxlv
Noel, Roden, xc
Northampton, Progress at, ccxvi
North-Eastern Hospital for Children, xix
Notes and Queries, viii, xix, xxix, xl, xlix, lx, lxviii, lxxviii, lxxxv, c, cix, cxx, cxxix, cxxxvii, clviii, clxvii, clxxx, clxc, cxcviii, ccix, ccxviii, ccxxiv, ccxxx, ccxxxviii, ccxliv, ccxlix
Novelties for Nurses, xv, lii, lxiv, lxxiv, cxxviii, clxv, clxxxvii, ccxxxii, ccxlii
Nurse Dolls, The, xcviii, ccxxxviii
Nurses and Medical Women, The Mutual Relations of, vi, xxvi
Nurses as Stewardesses, xcv
Nurses, District, lxxxvii
Nurses for Civilians in India, cviii
Nurses' Friends and Foes, xvi, xxiv, xxxiv
Nurses' Holidays, clxviii
Nurses (English) in Brazil, lxxxvi, xcvi, cxxiv
Nurses (English) on the Labrador, ccviii
Nurses, Old Time, ccxv
Nurses, Reserve of, clxxvii
Nursing, Ethics of, xiv, xxxv, xlvii
Nursing, History of, xxiv
Nursing, Technical Education Committees and, lviii
Nursing of Army Sisters, The, vii, xxxvi, lxxvii
Nursing in South Africa, ccxxxviii
Nursing in Chili, clxv
Nursing at Crieff, clxv
Nursing in Germany, cvii, cxvii, cxxxvi, clxxx, ccxxxvi
Nursing in Holland, ccl
Nursing in Ireland, lxxvii
Nursing on the Labrador, ccxxxvii
Nursing in New South Wales, lxxxiii
Nursing in Philadelphia, xxvii
Nursing in Switzerland, lxxxvi
Nursing in the United States, vii, clxvi
Nursing in the West Indies, lxv
Nursing of Diphtheritic Paralysis, xv
Nursing of Enteric Fever in Children, lxxxv
Nursing, On General:—
Catarrh, iii, xiii
Pneumonia, xxiii, xxxiii, xliii, liii, lxiii, lxxiii
Rheumatism, lxxxiii, xciii
Rheumatic Gout, ciii
Latent and Chronic Rheumatism, cxiii
Peritonitis, cxxiii, cxxxiii, cliii, clxiii
Typhoid Fever, clxxiii, clxxxiii, cxciii, cciii
Obstruction of the Bowels, ccxiii
Burns, ccxxiii
Nursing (Private) in Connection with Hospitals, cxvi, cxxxvii
Nursing Home at Kew, xiv
Nursing Service, The Indian, xxiv

Paris, Ambulance Work in, clxiii
Paris Letter, Our, xlvi
Pension Fund. *See Royal National*
Phosphorus (White) and Necrosis, clvii
Plague in Hong Kong, clxxvi
Poplar Hospital for Accidents, cvi
Practical Suggestions, lxxxviii
Presentations, xxviii, xlvi, lxvii, lxxxviii, cx cxiii, cxxxiv, ccxiv, ccxxx, ccxlii
Private Nursing in connection with Hospitals, On, cxvi, cxxxvii
Probationer's Night Watch, The, lxxvi

Queen Victoria's Jubilee Institute for Nurses, lvi, clviii, ccv
Queen's Nurse at Seacombe, cxxx

Reading to the Sick, For, x, xx, xxx, xxxix, l, lx, lxx, lxxviii, xcvii, cxviii, cxxvi, cxl, clx, clxvi, clxc, cxcviii, ccx, ccxvi, ccxxvi
Red Cross, The, clxxxvi
Royal Asylum, Morningside, Edinburgh, cx
Royal British Nurses' Association, xciii, cv; Annual Meeting, clxc
Royal National Pension Fund for Nurses v. Record Press, lv
Royal Patronage, Under, clxv

St. George's Hospital, Lectures at, v
Sark, A Holiday in, lvii
Seacombe, Queen's Nurse at, cxxx
Southend, clxviii
Spain, Eastertide in, iv
Spanish Hospital, A, xxviii
Stockton and Thornaby Nursing Association, l
Structure and Care of the Teeth, cxciv, cciv, ccxiv, ccxliii
Suggestions for Intending Probationers, clxxxiv
Switzerland, A Holiday in, clxxxviii

Technical Education Committees and Nursing, lviii
Teeth. *See Structure and Care of the*
Training, Thoughts on, lxvi
Two Hours Off Duty, cxxviii, cxxxviii
Typhoid and Maternity, xciv

Union, A Country, lxiii
United States, Maternity Work in the, lx
United States, Nursing in the, vii, clxvi
Universal Cookery and Food Association, The, clxiv

Victoria, Notes from, cxxxvi, clxvi, clxxxv

Waiting, ccxvi
Wants and Workers, vi, xix, xxiii, lvi, lxxiv, xc, cix, cxvi, cxxvii, clviii, clxviii, clxxvii, cxciv, ccxxv, ccxxxviii, ccl
Wardroper, The late Mrs., xxxviii
Wardroper, the late Mrs., Memorial to, xliv, lvi
Where to Go, viii, xix, xxix, xl, l, lvi, lxvi, lxxx, lxxxix, cviii, cxx, cxxx, clxviii, cxcv, cciv, ccxlii
Where to Go for a Holiday, xcviii
Where was the Label? ccxxiv
Winter in the Sunny South, A, ccix
Women Architects, ccviii
Women Inspectors, Qualified, xcvii
Women Volunteers, xxv
Women's Volunteer Medical Staff Corps, iv
Women's Work, viii, xxxvii, xlvii, lxiv, lxxiv, clviii, cxcviii, ccxliv
Workhouse Infirmary Nursing Association, xcvii
Workhouse Infirmary Nursing Association, Position of the, ccxv
Worthing, ccxxiv

Printed by W. H. and L. Collingridge, "City Press," 148 and 149, Aldersgate Street, London, E.C.

**Notice to Advertisers and Subscribers.**

All Advertisements, Orders for Copies of The Hospital, and Business Communications should be addressed to **The Publisher, NOT TO THE EDITOR**, The Hospital, 428, Strand, W.C.

The Cost of Subscription, post free, is as follows:—Per week, 2½d.; per quarter, 2s. 8d.; per half-year, 5s. 3d.; per annum, 10s. 6d. Foreign:—Per Annum, 12s. 8d.; payable, in all cases, in advance.

**Notices of Births, Deaths, and Marriages** are inserted in this column at a charge of 2s. 6d. for an announcement not exceeding four lines.

### NOTICE TO CORRESPONDENTS.

All MS., Letters, Books for Review, and other matters intended for the Editor should be addressed The Editor, The Lodge, Porchester Square, London, W.

The Editor cannot undertake to return rejected MS., even when accompanied by stamped directed envelope.

**"Burdett's Hospital Annual," 1894.**

Fifth year of publication. 900 pp. Boards, gilt lettering. A most complete guide to British, Colonial, Indian, and American Hospitals, Dispensaries, Nursing and Convalescent Institutions, and Asylums. Price 5s. Published by The Scientific Press (Limited), 428, Strand, W.C.

**NOTICE.—The Index for Vol. XIV. (ending Sept. 30th, 1893) is on sale at the Office of this Journal, price 2½d., post free.**

### Notes and News.

### Scraps and Gleanings.

A good balance is reported to exist on the funds of the York Dispensary.

Mons. Jules Simon having received a legacy of 100,000 francs from a stranger to him, has divided the sum amongst Paris charities.

An encouraging balance-sheet was presented at the annual meeting of subscribers to the Brecknock County and Borough Infirmary.

Dr. James Nicol, J.P., laid the foundation-stone last month of the new wing to the "Sarah Nicol Memorial Cottage Hospital" at Llandudno.

At the annual meeting of the Sidmouth Cottage Hospital Committee, the report for the year showed a serious increase in their adverse balance.

Miss Julia E. Kent, of Brighton, has bequeathed £100 to the Queen Charlotte Lying-in Hospital, and the same sum to the Sussex County Hospital.

Amended plans have been adopted by the Deal Town Council for the erection of the Infectious Diseases Hospital, the estimated cost of which was £1,700.

The annual meeting of the Victoria Home for Invalid Ladies, Headingley (Leeds), took place recently. A regrettable deficit of £165 is stated to exist in the accounts.

The Committee of the Newcastle Hospital for Diseases of the Skin again, at the close of another year, report on the continued prosperity and success of the hospital.

The working men of Evesham have shown their appreciation of the local Cottage Hospital in a practical manner, by again forwarding the year's subscriptions towards that institute.

The Duke and Duchess of Fife presided at the reopening of the old buildings of the St. Marylebone General Dispensary in Welbeck Street, which have been enlarged at a cost of £6,000.

The subscriptions to the proposed memorial to Professor Marcus Beck have come in so well that £800 has been handed over to University College to assist in endowing a bed in memory of the deceased.

Canon Blencowe has collected £20 towards the Children's Convalescent Home, West Kirby, which sum will be appropriated to supplying a cot in the institution to be appropriated to the use of his parish.

On Wednesday last week the Lord Mayor presided at the annual meeting of the Royal Hospital for Diseases of the Chest, City Road, E.C., and after inspecting the wards presented the hospital with ten guineas.

A halfpenny is subscribed weekly by each of Carey and Son's employés towards the Nottingham Hospital and kindred institutions, and the total amount derived from this source annually reaches a considerable sum.

The "Saturday" collection among the neighbouring colliers towards the Burton Infirmary, in spite of adverse times, proved to be a "record" collection of only a trifle under £1,000, £50 of which sum was handed over to the Nursing Institution.

The Baron de Hirsch has, through Mr. George Herring, most kindly sent £500 to the British Home for Incurables. This donation is to be placed to the fund for furnishing the new home, now being built at Streatham, and which will be opened in June next.

But for the receipt of legacy for £4,500 from the Rev. J. Spurrell, the committee of the Royal Alexandra Hospital for Sick Children at Brighton would have had a depressing report to lay before the governors at the twenty-fifth annual meeting which lately took place.

The Portuguese Government has authorised Dr. Eugenio Augusto Perdigao, Surgeon-Major in the 2nd Regiment of Cavalry in the Portuguese Army, to engage himself upon a history of military medicine in Portugal, a subject of which he has made a special study.

A handsome gift has been made to the Crieff Town Council, in the shape of an Infectious Diseases Hospital for the locality. Dr. and Mrs. Meikle are the munificent donors, and the hospital will consist of twenty-four beds, besides other accommodation, and it will cost close upon £4,000.

The report of the Norwich Eye Infirmary stated that 132 patients had been treated in the hospital as in-patients, and 403 as out-patients, during the year. Whilst the subscriptions were said to have fallen off somewhat, it was a source of satisfaction to the committee that legacies had increased.

The expediency of combating the prevailing epidemic in Bristol and the outlying districts continues to be much discussed in that locality. The Bristol Sanitary Authority has thirty vacant beds at the Norer's Temporary Hospital, some of which it is now proposed to utilise for the accommodation of cases from outside districts.

The formal opening of the extension of the Homœopathic Hospital at Bath has taken place. This hospital has extended its sphere of usefulness by opening a nursing institution and a new in-patient department. The new institution is a gift to the city from Miss Jennings, and is open to homœopaths and allopaths alike.

It has been proposed to meet a much-needed want at Plymouth, and erect a Maternity Hospital in that locality. The organizers of this scheme report that the expense of the undertaking need not necessarily involve any great outlay, whilst the completion of such a charity would confer a great boon on the female population.

The annual report of the Norwich Jenny Lind Infirmary for Sick Children states that the large number of 1,656 patients have been received for treatment during the year. The expenditure on the working of the institution for the twelve months has amounted to £1,264. The subscription list shows an increase on last year's. After all expenses had been met a balance of £54 to the good remains on the funds of the institution.

On the invitation of the Town Council, a meeting was recently held in the Council Chambers, Peterhead, to consider the claims of the Aberdeen Royal Infirmary on public sympathy and support. A considerable number of patients from Peterhead having benefited by the infirmary, it was proposed to establish some means of recognising the benefits thus received, and, amongst other suggestions, that of a Hospital Sunday at Peterhead received warm support.

### Medical Vacancies and Appointments.

### APPOINTMENTS.

W. Adam, M.B.Edin., of Rainhill Asylum, appointed Assistant Medical Officer to the Grahamstown Asylum, South Africa. H. Ainsworth, M.R.C.S., L.R.C.P., appointed House Physician to the Manchester Royal Infirmary. W. T. Atwood, M.R.C.S.Eng., appointed Ophthalmic Surgeon to the Torbay Hospital, Torquay. L. A. Baine, M.D.Durh., appointed Assistant Medical Officer to the Monsall Fever Hospital of the Manchester Royal Infirmary.

### VACANCIES.

#### Resident Medical Officers.

The following vacancies are announced this week, the amount of salary in each case being given after the name of the institution:

Bedford General Infirmary and Fever Hospital.—House Surgeon, doubly qualified. Salary £100 per annum, with apartments, board, lodging, and washing. Applications to the Secretary by April 10th.

Birmingham General Dispensary, Birmingham.—Resident Surgeon. Salary £150 per annum, with an allowance for cab hire, and furnished rooms, fire, lights, and attendance. Applications and testimonials to Alex. Forrest, Secretary, by April 11th.

Carlow District Lunatic Asylum.—Assistant Medical Officer, unmarried, and not more than 30 years of age, and doubly qualified. Salary £100 per annum, and emoluments valued at £100 per annum. Applications to the Medical Superintendent. Election on April 13th.

Cancer Hospital (Free), Fulham Road, S.W.—House Surgeon. Appointment for six months. Salary at the rate of £50 per annum, with board and residence. Applications to the Secretary by April 9th.

Liverpool Eye and Ear Infirmary.—House Surgeon, doubly qualified. Salary £80 per annum, with residence and maintenance. Applications to Reginald Haigh, Honorary Secretary, 13, Bevey's Buildings, George Street, Liverpool, by April 7th.

Oldham Infirmary.—Senior House Surgeon. Salary £80 per annum, with board and residence. The Junior House Surgeon is a candidate, and if elected there will be a vacancy for Junior House Surgeon. Salary £50 per annum, with board and residence. Doubly qualified. Applications to Harold Lees, Secretary, by April 10th.

Royal Hants County Hospital, Winchester.—House Surgeon. Salary £100 per annum, with board and lodging. Applications to the Secretary by April 11th.

Royal Hospital for Diseases of the Chest, City Road, E.C.—Resident Medical Officer. Appointment for six months, when re-election is required. Salary at the rate of £100 per annum, with furnished apartments and board. Applications to the Secretary by April 11th.

The Coppice, Nottingham.—Assistant Medical Officer; unmarried, and not more than 28 years of age. Salary £120 per annum, with furnished apartments, board, washing, and attendance. Applications to Dr. Tate at the Asylum by April 14th.

#### Non-Resident Medical Officers.

City of London Hospital for Diseases of the Chest, Victoria Park, E.—Pathologist. Salary 100 guineas per annum. Applications to the Secretary at the office, 24, Finsbury Circus, E.C., by April 12th.

Dental Hospital of London and London School of Dental Surgery, Leicester Square.—Demonstrator. Honorarium £50 per annum. Applications to Morton Smale, Dean, by May 14th.

Royal College of Physicians.—Milroy Lecturer. Applications to Edward Liveing, M.D., Registrar, by April 9th.

